# A systemic approach to assess the potential and risks of wildlife culling for infectious disease control

**DOI:** 10.1038/s42003-020-1032-z

**Published:** 2020-07-07

**Authors:** Eve Miguel, Vladimir Grosbois, Alexandre Caron, Diane Pople, Benjamin Roche, Christl A. Donnelly

**Affiliations:** 1 Medical Research Council Centre for Global Infectious Disease Analysis, Department of Infectious Disease Epidemiology, Imperial College London, London, UK; 2 MIVEGEC (Infectious Diseases and Vectors: Ecology, Genetics, Evolution and Control), IRD (Research Institute for Sustainable Development), CNRS (National Center for Scientific Research), Univ. Montpellier, Montpellier, France; 3 CREES Centre for Research on the Ecology and Evolution of Disease, Montpellier, France; 4 ASTRE (Animal, Health, Territories, Risks, Ecosystems), CIRAD (Agricultural Research for Development), Univ. Montpellier, INRA (French National Institute for Agricultural Research), Montpellier, France; 5 UMMISCO (Unité Mixte Internationnale de Modélisation Mathématique et Informatiques des Systèmes Complèxes, IRD/Sorbonne Université, Bondy, France; 6 Departamento de Etología, Fauna Silvestre y Animales de Laboratorio, Facultad de Medicina Veterinaria y Zootecnia, Universidad Nacional Autónoma de México (UNAM), Ciudad de, México, México; 7 Department of Statistics, University of Oxford, Oxford, UK

**Keywords:** Conservation biology, Ecological epidemiology, Infectious diseases

## Abstract

The maintenance of infectious diseases requires a sufficient number of susceptible hosts. Host culling is a potential control strategy for animal diseases. However, the reduction in biodiversity and increasing public concerns regarding the involved ethical issues have progressively challenged the use of wildlife culling. Here, we assess the potential of wildlife culling as an epidemiologically sound management tool, by examining the host ecology, pathogen characteristics, eco-sociological contexts, and field work constraints. We also discuss alternative solutions and make recommendations for the appropriate implementation of culling for disease control.

## Abstract (web summary)

Eve Miguel et al. discuss the potential and practice of wildlife culling to control infectious disease both in the wildlife population and in transmission to humans. They identify several factors that must be considered when using culling, including host and pathogen ecology and eco-sociological contexts.

## Introduction

Infectious diseases that can be transmitted both within and between wild and domestic animals and human host populations represent almost 75% of the infectious diseases that have (re-)emerged in human populations in the last century^1^. As these pathogens are typically transmitted to multiple host species^2^, wildlife are often an important component of such systems^3^, as it is illustrated by the novel coronavirus SRAS-COV-2 which is thought to have emerged in seafood markets in Wuhan, China^4^.

Infections from wild animals can be directly transferred to humans, as illustrated by Human immunodeficiency virus-1 (HIV-1), which causes acquired immune deficiency syndrome (AIDS) in humans as the result of an independent cross-species transmission event of simian immunodeficiency viruses (SIVs), which infect African apes^5^. Vectors can be the link between different populations (i.e., wild animals and humans). For instance, the yellow fever virus is transmitted from monkeys to humans by mosquitoes^6^. Wild animals can also transmit pathogens that are harmless to humans but harmful to domestic animals, with often significant impacts on animal health and agricultural economics, as exemplified by foot-and-mouth disease (FMD). Finally, wild animals and humans can also be linked through extended transmission chains via domestic animals, without vectors, as illustrated by influenza transmission through humans, pigs, and wild and domestic birds^7^. Although it was found that emerging infectious diseases were 4-fold more frequent in the 2000s than in the 1940s^8^, pathogen spillover among different species still remains a rare phenomenon that requires the fulfillment of different conditions^9^. Particularly, the interspecific interactions between donor and recipient species must be frequent enough and the pathogen has to be sufficiently pre-adapted to the recipient host species. Consequently, proximity is a key driver of pathogen spillover among species, especially for directly transmitted viral diseases^10^. Anthropogenic pressure on the natural environment has led not only to the fragmentation of natural habitats and the extinction of numerous species^11,12^, but also to an encroachment of farming and urban activities at the periphery of areas occupied by wild animal populations^13,14^.

As soon as the concept of pathogen transmission started to be understood, culling of domestic animals was put in place to manage animal diseases, due to its apparent simplicity. Indeed, it was used for the management of the first rinderpest epidemics in Europe in 1709 in addition to movement restriction, *cordons sanitaires*, quarantine, disinfection, and carcass burying^15^. Later, vaccination campaigns resulted in the successful eradication of rinderpest^16^. The objective of animal culling is to reduce the rate of infectious contacts below the threshold required for pathogen persistence by decreasing the number and density of infectious and susceptible animals^17^. More recently, culling of cattle, sheep, and pigs was implemented to control the FMD epidemic in the United Kingdom (UK) in 2001^18^, of poultry to limit the transmission of highly pathogenic avian influenza viruses in 2005^19^, and of pigs to control the African swine fever epidemic in China in 2018^20^. During the 2001 FMD epidemic, 4 million domestic animals were culled for the purpose of disease control, and at least a further 2.5 million animals were destroyed in ‘welfare’ culls^21^. The very high economic cost and wider impacts on society of this culling strategy raise the question of the best approach to adopt in future epidemics.

Wildlife culling also has been used for disease control. During the 20th century in southern Africa, culling was implemented to create wildlife-free corridors around national parks to protect cattle farms from the spread of trypanosomiasis^22^. About 660,000 animals from 36 species, including large mammals such as black rhinoceros (*Diceros bicornis)* and elephants (*Loxodonta africana)*, were killed^23^. Rabies control in Europe included red fox (*Vulpes vulpes*) depopulation schemes^24^ that were undertaken from the 1960s, with limited success compared with oral immunization^25^. More recently, culling has been used to reduce populations of European badger (*Meles meles*) in the UK and the Republic of Ireland, of brushed-tailed possum (*Trichosurus vulpecula*) in New Zealand, and of African buffalo (*Syncerus caffer caffer*) in South Africa because these species were infected with *Mycobacterium bovis*, the zoonotic pathogen responsible for bovine tuberculosis (Table 1).

**Table 1:** Examples of culling strategies undertaken in recent decades (i) zoonotic bovine tuberculosis (bTB) in wildlife which can affect domestic animals and humans: and (ii) Devil Facial Tumour Disease exclusively in the Tasmanian devil.

Multi-host pathogens and culling: example of bovine tuberculosis (bTB)						
Pathogen	Host species	Area	Period	Type of culling	Success/problems	References
Bovine tuberculosis	European badger	Avon, UK	1975–1980	Non-selective	- (***) Apparently reduced cattle bTB	^144,172^
		Ireland	1989–2005	Non-selective & reactive	- (***) Longer survival time to the next bTB episode for cattle	^173^
		Ireland	1997–2002	Non-selective & proactive	- (***) Decrease in herd restriction	^174^
		RBCT^a^, UK	1998–2005	Non-selective & proactive	- (***) Decrease in TB cases for cattle inside culling area but increase outside	^37,100^
					- (*) Increase in *M. bovis* prevalence in culled badgers in later culls	
		RBCT^a^, UK	2000–2003	Non-selective & reactive	- (*) Increase cattle bTB within reactive culling areas	^38,100^
					- (*) Increase in *M. bovis* prevalence in culled badgers in later culls	
	African buffalo	South Africa	1999–2006	Selective: test & cull	- (***) Disease hotspots did not expand spatially over time	^175^
	Feral water buffalo	Australia	1976–1997	Widespread non-selective culling (close to elimination)	- (***) bTB eradication, but no other wild species involved in transmission	^176^
	Brushed-tailed possum	New Zealand	Start in 1972	Non-selective & widespread culling + systematic «overkill»^b^ since 2000	- (***) Considered as a pest: progress toward elimintion of bTB in cattle since 1994 with bTB management in cattle	^177,178^
	Wild boar	Spain	2000–2011	Non-selective	- (***) Prevalence decrease in wild boar and potentially in sympatric red deer, but culling occured only in 3 sites (*)	^179^
			2007–2012	Non-selective & high hunting pressure	- (***) bTB prevalence decreased in fallow deer, but not homogeneously: in the last season of study there was an increase in bTB-infected male animals and bTB prevalence remained high in the wild boar population (*)	^180^
	Wild boar + deer + badger	France	2006	Non-selective & red deer elimination and widespread culling of wild boar & badger	- (***) First cases detected in wild animals in 2001. No cattle breakdown until 2015. Recent outbreaks in cattle and case detection in wild boar (2016) (*)	^39^
	White-tailed deer	United States	2005–2010	Non-selective widespread hunting + ban feeding	- (***)bTB prevalence decreased from 1.2% in 2005 to undetectable level in 2010	^181^
			2007–2008	Selective: test & cull	- (*) bTB prevalence was slightly lower than expected. The cost (US$ 38000 /per positive animal) and efforts resulted in an unfeasible management strategy	^182^
**Single-host pathogen and culling: example of devil facial tumor disease (DFTD)**						
DFTD	Tasmanian devil	Tasmania	1999–2008	Selective culling on infected symptomatic individuals	- (*) Selective culling of infected individuals neither slowed the disease progression rate nor reduced the population-level impacts of this debilitating disease	^29^

The table summarizes the species culled, the area, the period, the type of culling strategy used and the main conclusion. (***) indicates that the culling strategy had a beneficial impact and (*) a detrimental impact. ‘Non-selective & reactive’ culling implies that the culling strategy targets wild individuals near the infected individuals, in contrast to proactive where all wild animals are targeted in a defined area.

^a^RBCT: Randimised Badger Culling Trial.

^b^Possum numbers are reduced to well below the model-predicted threshold for bTB persistence.

Currently, the conclusions of the final report by the Intergovernmental Science-Policy Platform on Biodiversity and Ecosystem Services suggest that around 1 million species already face extinction, and many more within few decades^26^. This implies an increasingly parsimonious management of wildlife. In many settings, culling is no longer considered an acceptable policy option for disease control because it significantly affects biodiversity conservation and more generally ecosystem functioning^27^. Moreover, removing wild animals from natural populations can have unexpected counterproductive consequences on pathogen transmission within the host community. Finally, depending on the species targeted for culling (e.g., protected, pet or livestock species), the public response to culling-based control options can facilitate or hinder their implementation. Consequently, the cost-effectiveness and cost-benefit balances of some wildlife culling options is now a topic of intense debate among scientists, policy makers, stakeholders, and the general public (Table 1). In this review, we assess the evidence regarding wildlife culling as a potential control strategy in several epidemiological contexts, compared with other available control options (see Supplementary Fig. 1, Table 1 and the Supplementary Information for article selections from 1992 to 2018). We describe socio-ecosystem and infectious disease dynamic features that must be understood in order to design effective culling policies. Particularly, we review the range of potential consequences of culling, including its counterproductive effects on the disease system. Finally, we discuss wildlife culling relative to alternative disease control policy options.

## Ecological, epidemiological and eco-sociological aspects of wildlife culling strategies

**Fig. 1:**
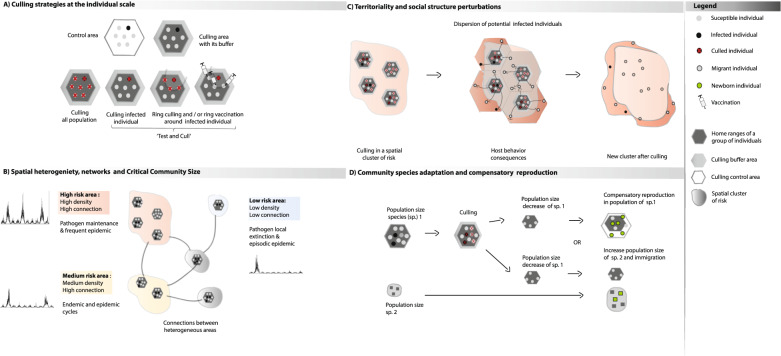
Culling strategies at the individual and population scales and culling response prediction. **a** The most common culling strategies used to manage a disease in wild populations in theoretical conditions (see Box 1). A buffer around the culling area is often defined to alleviate edge effects, for instance, the impact of survivors migrating outside the culling area^75^. The size of the culling and buffer areas together is the same as that of the control area. Depending on the diagnostic capacity and capture success, potentially all individuals or only the infected individuals are eliminated in the culling area (see Box 1). In the second case, the individuals potentially in contact with each detected infected individual could also be culled, thereby generating a “cordon sanitaire”. Culling is occasionally complemented with vaccination. **b** Spatial heterogeneity can result in the appearance of epidemiological risk clusters. It is usually considered that the risk of pathogen maintenance is higher in a high-density sub-population well connected with other sub-populations (see Box 1). Such a cluster can function as a reservoir that can maintain a pathogen and infect other sub-populations. Less dense and connected sub-populations are more likely to show stochastic epidemiological dynamics where phases of local pathogen extinction alternate with epidemic phases and possibly endemic phases^118^. These parameters help to prioritize the spatial and temporal risks and consequently to determine the best areas and periods for culling and the proportion of individual to remove for a successful culling strategy. **c** After culling in a high-risk cluster, the surviving hosts may migrate outside their home ranges into new territories, or may increase their home ranges. As surviving hosts may be infectious, such responses to culling can increase the risk of pathogen diffusion. **d** Culling individuals of a target species can affect the population demography. Compensatory reproduction can be observed in the target species. The reproductive parameters of competitive species may also be affected through the ‘release’ of an ecological niche.

The design of a culling strategy requires the identification of the species and individuals to be culled as well as the spatial and temporal extent of the culling. Culling can have various forms, from the most extensive (i.e., culling the whole target population)^28^, to the most selective (i.e., removing only the infected individuals; i.e. test and cull)^29^ (Fig. 1a and Box 1). Such choices should be informed by an evidence-based understanding of the focal eco-epidemiological system (Fig. 1).

Box 1 Glossary Culling strategies

To assess whether culling significantly affects disease dynamics, comparison areas needs to be identified and followed simultaneously^119^. In many case studies reviewed here, comparison areas were not monitored, and the culling action effectiveness was simply based on the prevalence rates in the susceptible species (including the culled species) before and after the action.

Culling an entire population:

Culling all individuals in populations of one or several species identified as maintenance community in a defined area (i.e., island, country or region) is one option to reduce the disease risk. In ring culling strategies (Fig. 1a), the culling effort is most intense in high-risk areas and decreases with the distance from the high-risk area. The effectiveness of such strategies relies on the availability of good quality spatial data on disease risks^120^. Several options are considered for wildlife culling (e.g., gassing dens, cage trapping, snaring and poisoning); however, each method increasingly needs to be evaluated in terms of animal welfare, and options that guarantee minimal stress and suffering should be favored. Gassing badger setts with cyanide is now considered inhumane, and anoxic gas or gas-filled foam is preferred^121^. When using cage trapping, animals should not be left in traps for too long. Gunshot can cause instantaneous death, but its use needs skill and precision^35^.

Test and cull:

The test-and-cull strategy (Fig. 1a) is frequently used for domestic tagged populations, and rarely for wild animals. Its aim is to remove only the infected individuals from the targeted population. Its potential success depends on the accuracy of the available diagnostic tests. If a portable and rapid test is not available to detect infections in the field, individuals will need to be held until the test result is available, or to be identified for allowing the re-capture of the positive ones^29,96,122^.

Critical community size and metapopulation (Fig. 1b):

The critical community size is the minimal size of a closed host population where a pathogen could persist indefinitely^123^. A metapopulation is a network of spatially distinct sub-populations of a species connected by individual movements. Important types of metapopulation models include the *‘continent-island’* and *‘source-sink’* models, where all migrants originate from a large spatial unit (*‘continent’ or ‘source’*) and colonize smaller units (*‘island’ or ‘sink’)*^124^. Metapopulation models are used to investigate the epidemiological dynamics of structured host populations^125,126^.

How does transmission vary with host density?

The two extreme scenarios are:

Density-dependent transmission:

Density-dependent transmission occurs when the rate at which a susceptible individual experiences contacts with infectious individuals increases with the density of infectious individuals^127^. Highly contagious diseases, such as foot-and-mouth disease^128^, display density-dependent transmission.

Frequency-dependent transmission:

Frequency-dependent transmission occurs when the rate at which a susceptible individual experiences contacts with infectious individuals increases with the frequency of infectious individuals in the host population. The transmission of sexually transmitted and vector-borne diseases is often frequency dependent^129,130^.

Epidemiological systems rarely conform strictly to either of these two extremes. Most often, they fall somewhere on a continuum between density- and frequency-dependence, or can conform to both. Some systems are characterized by density-dependent transmission at low host densities, and by frequency-dependent transmission at high host densities. Some other transmission modes could exist^127^, such as vertical transmission^131^.

Non-selective culling reduces host density, but not the frequency of infectious individuals. Therefore, culling is not expected to reduce the transmission risk if transmission is frequency-dependent because such risk depends only on the proportion of infectious individuals in the population^127^. However, the absolute incidence of new infections will decrease due to the decreased number of susceptible individuals.

Methods to estimate population density or abundance

Abundance is the number of individuals of a given species in a population, while density is the number of individuals of a given species per unit of area.

Numerous methods, models, and tools have been developed by ecologists to estimate population density or abundance by taking into account observation probability and its variation according to factors, such as time of day or season^132^. These include distance sampling methods to analyze line transect data^133^, capture-recapture methods to analyze individual scale monitoring data^134^, and point-transect methods to analyze data generated by camera traps (i.e. capturing pictures, video, infrared or not) deployed on grids within a study area^135^.

## Hosts

### Determining the target species

The identification of the species involved in disease transmission is an important step for designing control strategies. Pathogens can have narrow animal host ranges, such as the Middle East respiratory syndrome (MERS) coronavirus that emerged in humans in the Middle-East in 2012 and that circulates, based on the current knowledge, only in dromedary camels^30^. Others can infect a broad spectrum of animal hosts, such as *M. bovis* (see Table 1 and ref. ^31^), which can infect a wide range of even-toed ungulates. When the host range is broad, culling only one host species can produce unexpected effects. In this situation, it is important to identify the competence (the ability to maintain and transmit infections) of each host community, and to adjust the culling strategy accordingly.

Johnson et al. gave an excellent empirical demonstration of the role of the host community, species richness, and host competence in disease transmission^32^. They showed that host diversity inhibits the virulent pathogen *Ribeiroia ondatrae* and reduces amphibian disease (78.4% decline in transmission in richer assemblages). In a different system, Dearing et al. showed that the Sin Nombre virus prevalence in deer mice is related to the mammalian community complexity. The prevalence was lower in more diverse communities, where deer mice had fewer intraspecific interactions, than in less diverse communities^33^. Failure to capture the complexity of the host epidemiological community can jeopardize disease control. Large-scale proactive and localized reactive culling strategies were implemented only in badger populations to control the *M. bovis* spillover to cattle in the UK, although other wild species present in these ecosystems, such as deer, are sensitive to the disease and can excrete the bacteria^34^. This control strategy gave mixed results (see Table 1 and Boxes 1 and 2 to understand these findings, and refs. ^35–38^). Therefore, after the detection of the disease in France in wild ungulates in 2001 and in cattle in 2002, a multi-host culling approach that included deer, wild boars, and badgers (Table 1) was adopted to limit the spread of bovine tuberculosis in wildlife^39^. This example highlights the importance of determining the role of each influential host species for understanding the transmission system epidemiology^40,41^. Within a community, different species interact in a geographic area and have interdependent densities, according to ecological processes (e.g., predation, competition, and parasitism)^42,43^. If a species is weakly connected inside the community (i.e., contact rates and disease competence are low), the impact of its removal on the ecological functions of other species will be limited. However, the ecological niche released by this removal may make available resources for competitive species^44^ that may be more connected and/or competent. Conversely, reducing the density of a highly connected species could disturb the whole ecosystem (e.g., keystone species^45^) with unpredictable epidemiological consequences.

Box 2 Case study of the eradication efforts of endemic pathogens at the wild/domestic interface: veterinary, public health, and conservation issues *Mycobacterium bovis*: cattle and badger (*Meles meles*) populations—United Kingdom

In the late 1960s, bovine tuberculosis (bTB), caused by *Mycobacterium bovis*, in cattle was almost eradicated from the United Kingdom (UK)^136^. Programs based on systematic skin testing of cattle herds and compulsory slaughter of test reactors, movement restriction of known infected herds, and slaughterhouse surveillance led to a dramatic reduction of bTB^137^. However, recently, bTB has spread from the southwest of England to large areas of England and Wales^138^, and is now endemic in some regions (southwest and parts of central England, and southwest Wales) and sporadic elsewhere^139^. The cost of bTB control in the UK is over £100 million per year^140^. Cattle movements are the predominant contributory factor to bTB spread in areas outside the traditional disease hot spots^138^; however, its nation-wide spread is also explained by other factors, such as farm management practices (herd size, restocking, farm type)^141^.

The sources of *M. bovis* infection in cattle are multiple and poorly quantified (i.e., who infected whom among species^142,143^). In some high-risk areas of the UK, *M. bovis* infection is common in badgers, widely believed to constitute the main wildlife reservoir in the UK^35,144^. The potential role of other wild mammals, such as deer or other carnivores, is not clearly understood. A study on bTB infection in wild mammals in the South-West region of England confirmed infections in fox *(Vulpes vulpes)*, stoat *(Mustela ermine)*, polecat (*Mustela putorius*), common shrew (*Sorex araneus)*, yellow-necked mouse (*Apodemus flavicollis)*, wood mouse *(Apodemus sylvaticus)*, field vole *(Microtus agrestis)*, grey squirrel *(Sciurus carolinensis)*, roe deer *(Capreolus capreolus)*, red deer *(Cervus elaphus)*, fallow deer *(Dama dama)*, and muntjac (*Muntiacus reevesi*)^34^. Despite the substantial infection prevalence in these host species, their competence for maintaining *M. bovis* in the population (as maintenance community) remains poorly assessed. However, the results suggest that deer should be considered a potential, although probably localized, source of infection for cattle^34^.

Badgers have been increasingly implicated in the persistence and re-emergence of bTB over the last 40 years^87^. The Randomized Badger Culling Trial, undertaken from 1998 to 2005^35^, demonstrated that culling could both increase and decrease the disease risks for cattle, depending on the culling spatial scale and the cattle location relative to the culling area^37,38^. As culling reduced not only badger density, but also increased the movements of surviving individuals, it modified the within-badger contact network. After culling, the infection prevalence increased in badger populations^99^. In cattle, the incidence of confirmed bTB decreased by 23% among cattle herds inside the proactive culling areas^37^, but increased by 25% in the vicinity of the proactive culling areas^37,71^. Moreover, the long-term reduction in badger populations following culling raises wildlife conservation concerns^74^. Despite protests and legal challenges, culling is still implemented in the UK and has been expanded also to low-risk areas, when originally it was focused only on high-risk areas^145^.

### Assessing the population size

If the target host population is large, it may not be possible to cull enough individuals to limit pathogen transmission. This could apply to large bat colonies of central Africa, where they are suspected of being reservoirs for the Ebola and Marburg viruses^46,47^, and of Latin America, where bats can transmit rabies^28^. When the southwestern United States and Mexico are considered together, the census population size of the roosting colonies of Mexican free-tailed bats (*Tadarida brasiliensis mexicana)* may easily reach 10^7^–10^8^ individuals^48^. Conversely, if the host population is very small, culling might generate conservation concerns (Box 3). Population dynamic models can be used to predict whether culling could lead to a population crash.

Before culling actions are undertaken, size estimation of the target population must be combined with an evaluation of the culling rate required to decrease the prevalence or to eliminate the pathogen in order to determine the number of individuals that should be culled. At this stage, it is important to account for uncertainty in the size estimation of the target population. For example, to control bovine tuberculosis in 2015, licensees in Dorset county (UK) were required to cull at least 615 badgers, which corresponded to the recommended 70% culling rate^49^ for the lower bound of the 95% confidence interval for the size estimation of that population (879–1547 animals). However, if the actual population size were close to the upper bound of the estimation, culling 615 badgers would have resulted in a reduction by 39.8% of the population size, which would not have been sufficient to control the spread of bovine tuberculosis in cattle. Numerous methods, models, and tools have been developed by ecologists to estimate population abundance (see Box 1 for more details).

Box 3 Case study on the eradication of emergent pathogens in wildlife, a conservation issue Emergent pathogens are a threat to domestic and/or human populations and also to wildlife populations and their conservation^29^. The recent epidemic of the Peste des petits ruminants virus in 2017 in Eastern Asia in the Saiga antelope (*Saiga tatarica Mongolica)* is a perfect example with a mass mortality affecting the two-third of the endangered species population^146^. More examples of pathogens affecting wildlife species (i.e. Canine distemper virus in the Serengeti ecosystem), and the control by culling (i.e., Chronic Wasting Disease in North America) in Supplementary Information.

Tasmanian devil (Sarcophilus barrisii)—devil facial tumor disease (DFTD)—Tasmania

The Tasmanian devil, the largest surviving marsupial carnivore, is threatened by DFTD, an emergent cancer that was first detected in Tasmania in 1996. DFTD is easily detectable as large tumors on the animal’s face (cf. picture). Transmission occurs via the transfer of live tumor cells from an infected to a healthy devil^29^. Death usually occurs after 3–6 months with no recovery and no immunity. Resistance is rarely recorded^147^. The disease affects most individuals, leading to a population decline of 60-90%, depending on the region^148^. In the region where the disease was first reported, the mean spotlight sightings declined by 80% from 1993–1995 to 2001–2003^149^.

It was suggested that selective culling of infected hosts was possible and might limit or even stop the disease spread^29^. As infectious contacts typically involve bites during sexual interactions, transmission is frequency dependent. Consequently, selective culling of infected individuals, rather than a non-selective reduction in Tasmanian devil population density, might reduce transmission. The culling of infected animals is generally publicly acceptable and should have limited impacts on population dynamics because the life expectancy of infected individuals is very short^29^. Another feature that might contribute to the success of selective culling is that the devil seems to be the only host species of the disease^149^. Furthermore, Tasmanian devils are easy to capture. Yet, comparison of the disease progression in an area where selective culling was implemented and in a control area (no culling) showed no decline in DFTD prevalence after 2.5 years of selective culling^29,77^. Many of the demographic changes and epidemiological patterns indicative of disease impact (change in the population age structure, decline in adult survival rate, increase in infection rates, decline in population size) occurred more rapidly in the area subjected to culling. Possible reasons for this failure include the existence of cryptic sub-populations that are impossible to catch and may act as reservoir populations, and immigration of infected individuals from areas without culling^29^.

Moreover, transmission modeling indicated that culling every 3 months would not be sufficient to suppress the disease even in isolated populations. More frequent removal might be more effective, but this culling rate might not be achievable^77^.

### Identifying the individuals to be culled

In the simplest theoretical case, each infected individual in a population is equally likely to transmit the infection. In reality, the risk of onward transmission between individuals even within a particular species can greatly vary (e.g., super-spreaders in host populations)^50,51^. Such heterogeneity can result from individual characteristics, including immune status and behavior^52^. Selective culling strategies can target individuals on the basis of their infection status (e.g., targeting only infectious individuals) or their demographic characteristics, such as age or sex (see Box 1 and Fig. 1). An important and sometimes overlooked aspect of culling for disease control is the identification and prioritization of populations or individuals that have the greatest potential to transmit the infection^53^. In reality, the culling rate is often higher for individuals that are easier to capture and/or kill. When such individuals are also more likely to be immune and resistant to the disease, this can interfere with the establishment of herd immunity, which is a natural barrier to transmission^29,54,55^. This is the case of culling campaigns to control the classic swine fever in wild boar where old individuals that are often immune and resistant are more easily eliminated^56^.

### Understanding spatial distribution and connectivity

The habitat of a wild animal population is often fragmented, either naturally, or as the result of human-driven modifications of the landscape^57^. The populations occupying such habitats are structured in sub-populations that may be connected through dispersal. The dynamics of the resulting meta-populations are governed by local dynamics and also by the colonization and extinction processes that determine patch occupancy. In fragmented wildlife populations, the culling objective could be to reduce the size of sub-populations below the critical community size, which is the minimum population size required to maintain the pathogen^58,59^ (see Box 1 and Fig. 1b). However, a pathogen can become extinct within some sub-populations^60^, but may persist in other cryptic sub-populations that are impossible to cull and can play the role of reservoir^29^. In the case of Tasmanian devil culling, such cryptic sub-populations may have acted as reservoir for immigration of infected individuals from areas without culling in Freycinet peninsula on Tasmania east coast, between 2004 and 2008^29^.

## Pathogens

### Understanding transmission dynamics

For several infectious diseases in wild animal populations transmission increases with host density^61^. For other infectious diseases, the transmission risk depends on the frequency rather than on the density of infectious hosts^62^ (e.g., sexually transmitted diseases in humans^63^). Intra- and inter-specific transmission also could be differentially influenced by density and frequency, and, therefore, this aspect needs to be thoroughly characterized. Understanding whether transmission is frequency- or density-dependent (see definitions in Box 1) is important for the development of an efficient culling strategy. Indeed, only culling strategies that target infectious individuals or individuals that are most likely to become infectious would be efficient for the control of frequency-dependent diseases. Conversely, untargeted culling strategies might be efficient for density-dependent diseases. However, whether disease transmission is density or frequency dependent can be difficult to determine^29,64^. The analysis of epidemiological and genetic data combined with mechanistic models allows testing different transmission scenarios (e.g., density-dependent, frequency-dependent and/or environmental transmission) and can provide valuable insights into pathogens dynamics^65^.

Furthermore, the structure of the underlying contact network is affected by the host ecology, the pathogen species, and the spatial structure. Consequently, there is often considerable uncertainty on the transmission risks within a given eco-epidemiological system. For instance, it was hypothesized that rabies transmission in vampire bats in southern America was density-dependent due to the large size of the colonies. However, an empirical study showed no relationship between host density and seroprevalence^28^. Even in large colonies, any single animal has a limited number of neighbors to bite, and infectious contacts may not be homogenous due to the social structure within the colony. In Peru, the rabies virus might be maintained in bats through frequency-dependent transmission with a key role for juvenile and sub-adult bats, leading to the observed inefficacy of culling to eliminate transmission within bats and also in humans and domestic animals^28^. In discontinuously distributed host populations, frequency-dependent transmission and endemic local dynamics in some sub-populations can coexist with density-dependent transmission and epidemic local dynamics in other sub-populations, as observed for rabies in foxes^66^. A recent study on rabies in Tanzania highlighted the importance of the environment (e.g., roads, rivers, elevation, and dog density) besides epidemiological and genetic data. This combined approach showed that the localized presence of dogs was the most important determinant of rabies diffusion, and not their density. This implies that culling is ineffective for rabies control^67^.

Finally, indirect pathogen transmission (i.e., environmental transmission^65^) is often overlooked in disease management, particularly when planning culling strategies, despite the fact that it might limit the effect of culling on transmission. When pathogens can survive in the environment, they might persist for longer and their control is more difficult to achieve. Environmental transmission is most likely involved in the failure to control chronic wasting disease (CWD) in deer populations in America^68^ (see Supplementary Information—case studies), and might also explain why, in America, brucellosis persists despite the very small population size of bison *(Bison bison)* (e.g., less than 200 individuals), one of its reservoirs^69^ (see Supplementary Information—case studies).

## Space and time

### Determining spatial-temporal scales

Most often, the population targeted for culling occupies a large territory and the geographic scope of the culling action must be specified. The locations of culling and non-culling areas (see Fig. 1a and Box 1 for definition) have significant consequences on the disease spatial distribution and transmission dynamics^70^. Hot spots of disease transmission are typically prioritized, although the establishment of a transmission firebreak might require culling also at some distance from the high incidence areas. Conceptually, in order to robustly estimate the culling impact, similarly structured areas (for instance, in terms of disease risk, animal populations, vegetation, altitude, temperature, precipitation) need to be identified and monitored. The immediate surroundings of a culling area, often called the buffer area, also should be taken into account for measuring the impacts of culling^71^ (Fig. 1a). For instance, the Randomized Badger Culling Trial in UK (Box 2) showed that repeated, widespread culling could reduce bovine tuberculosis incidence in cattle over 100-km^2^ areas, but that its incidence increased in areas immediately surrounding the culling areas^37^.

Regarding the temporal scale, empirical evidence suggests that culling may be effective as a short-term response to highly localized disease outbreaks^72^. However, a temporary use of culling has rarely produced significant long-term effects^73^. For instance, in culled areas of the UK, the badger population was back to pre-cull densities after about 10 years^74^.

### Defining culling rate and periodicity

In addition to spatial-temporal scales, the target culling rate and periodicity have to be considered when developing a culling strategy^64^. For this, approaches relying on epidemiological modeling are often used. Depending on the initial prevalence and incidence rates in a population, different scenarios are modeled by varying parameters, such as the population reduction rate and the cull duration and frequency. The model that maximizes the likelihood of pathogen local extinction is selected^75^. However, such approaches are typically hampered by the limited knowledge of the eco-epidemiological system and transmission processes^76^. For instance, models often consider that host and pathogen populations are well mixed, with homogeneous disease transmission among susceptible and infected individuals. However, it is now well known that transmission may vary over time, in relation to social structures, or depending on individual characteristics, such as age. Also, modeling can lead to the conclusion that an efficient culling strategy cannot be implemented. For instance, modeling studies on Tasmanian devils and devil facial tumor disease (DFTD) (Box 3) showed that culling every 3 months was insufficient to suppress the disease in isolated populations. More frequent culls are more likely to be effective, but the required target culling rate might be too high to be achievable^77^, or could threaten the host population survival.

## Socio-economic context

### Quantifying the necessary economic resources

Often, control strategies to manage disease reservoir populations are designed from an eco-epidemiological perspective, but ignore the economic trade-offs^78^, despite their importance, especially in low-income settings^79^. From a purely economic perspective, culling should be undertaken whenever cost-benefit analyses show that increased revenue from reduced disease risks exceeds the (direct and indirect) costs of culling, and culling performs better in the cost-benefit analyses than other disease control strategies^80^. However, cost-benefit evaluations often reveal that culling strategies are not economically sustainable. For instance, the quarterly culling of the Tasmanian devil population in the framework of DFTD control cost 200,000 Australian dollars per year per 100 km^2^ over almost 3 years without significant decrease in pathogen prevalence (see Box 3)^77^. More frequent removals would be required, but they are hardly achievable economically. For each culling strategy, the cost-benefit balance needs to be assessed over a range of culling rates and periodicity options. Such assessment might reveal that the ‘do nothing’ option is often better in terms of cost-benefit^81^. Haydon et al. examined the role of mathematical models in the implementation of approaches that went beyond the traditional control strategies of the 1960s (i.e., movement ban, disinfection of infected properties, and rapid slaughter of all animals within a flock/herd) to control the 2001 outbreak of FMD in the UK^21^. The direct and indirect economic costs of the 2001 epidemic are estimated at £3 billion and £5 billion, respectively. This, together with the widespread public concern at the visible slaughter of millions of animals, prompted a major revision of outbreak contingency planning^21^. For badger control, the Department for Environment, Food and Rural Affairs in the UK estimated a cost of £16,087,000 from 2013 to 2017^82^ (see Box 2 for epidemiological context and results). England has recorded 3888 new tuberculosis bovine cases in 2017^83^, compared with fewer than 100 per year in the 1980s^35^, with a total of 31,773 animals slaughtered, and a 7% increase year-on-year^83^.

### Exploring culling social acceptance

Wildlife culling to decrease disease incidence in domestic animals is often not culturally acceptable, and its acceptability may vary considerably among ethnic groups^69^. Alternative options to wildlife culling (see Box 4) are increasingly discussed in ethical committees in terms of animal welfare^84^. The citizens’ feelings toward the targeted species could also limit the success of culling-based control strategies. For instance, badgers are a protected species in the UK and are highly regarded by much of the population^85^. Badger culling has triggered vigorous protests in the UK^86^. In the Republic of Ireland, the use of restraints (snares) to avoid dispersion of animals from culled areas led to a more cost-effective badger culling, but it is considered unacceptable for animal welfare in the UK^87^ (see Box 2). Societal perceptions also affected the use of culling strategies to control brucellosis in North America (see  Supplementary Information—case studies). Elk, the main reservoir for brucellosis^88^, are generally appreciated and allowed to roam outside national parks with very few restrictions. Conversely, bison are less valued and they are slaughtered outside conservation areas, although they are a less infectious reservoir^55^.

Box 4 Alternative strategies to culling

1. Vaccination of healthy and at risk individuals in high-infection-risk areas can be implemented in addition to culling (Fig. 1a). The aim of this complementary strategy is to create an immunity barrier to reduce the transmission risk from infected areas outwards^69^. The key challenge is to achieve a sufficiently high coverage rate to have significant epidemiological effects, despite the limited economic and human resources. Vaccination is an efficient control strategy^150,151^ that raises fewer ethical and welfare issues compared with culling^66^. However, it is often difficult to obtain effective and easy-to-use vaccines for wild reservoir populations. The trade-off between the advantages and disadvantages of capturing a high fraction of a population is questionable, particularly if the conferred immunity is not long-lasting. For instance, a vaccination trial in brushtail possums (*Trichosurus vulpecula)* in New Zealand estimated that the antibodies against *Mycobacterium bovis* persisted only for one year^152^. Vaccination by oral ingestion of baits is an alternative to vaccination by injection in some settings. It is a generalist method (compared to injection) for immunizing a wildlife population^153^. Moreover, baits have to be eaten by a suitable fraction of susceptible animals, particularly if the required coverage level is high. For instance, a vaccine coverage of 70% is usually required for rabies. This strategy worked in Europe in the 1970s for rabies control in fox populations, following culling failure^17,154^. However, oral immunization poses four major challenges: (i) vaccine survival in the bait, (ii) the bait should attract only the targeted species^155^, (iii) unexpected consequences for species other than the targeted species in the same community that can ingest the baits, and (iv) difficulty to evaluate its efficiency^156^. Knowing the ecology and the behavior of the target host species is crucial for the success of oral immunization, where acceptance and palatability are key success factors^155^. In a vaccination strategy to control classical swine fever, 50% of young wild boar did not ingest the bait^157^ because older animals, which may have antibodies from an older infection, are often more aggressive in ingesting baits than younger individuals that are more susceptible to the disease^46,156^. These challenges have led some to conclude that depending on the ecology of the targeted host species, culling might be an easier control strategy.
2. Fencing off an area with the aim of decreasing the spatial overlap between populations (e.g., infected vs susceptible) by controlling their dispersion^158^ is particularly suited when infected hosts occupy defined areas (e.g., national parks). This strategy was frequently applied in southern Africa to decrease land use overlap between wild and domestic species^159^, but can have detrimental ecological effects by limiting migration and gene flow. For example, the fences placed by the beef industry in Botswana interfered with the wildebeest population’s seasonal movements and water access in dry years, leading to a decline from 315 000 to 16 000 individuals in the Kalahari ecosystem^23^. Moreover, although fencing can decrease the risk of pathogen transmission between populations, it could exacerbate it within the contained infected populations due to the increased density and contact rates in a restricted space^60,160^. Furthermore, the economic costs of fencing are high: 20,000 US $/km in Kenya for fences surrounding the Aberdare National Park, and 7250 US $/km for predator-proof fencing in South Africa with an additional maintenance cost of 3200 US $/km/year^161^.
3. Zoning within a country, in which high-risk regions (i.e., presence of wildlife maintenance communities) are isolated and/or fenced, is another strategy^159^. Connections between these areas and other parts of the country are limited and a special effort is directed toward surveillance in buffer areas. Zoning may limit geographical spread when trade and animal movements often are a major determinant of disease diffusion^138,162^. Some countries adopt regionalized zoning strategies to control a disease because free trade agreements can apply to the disease-free subnational regions^163^. For instance, three areas are considered in England for bTB testing: current high-risk area with annual testing, current edge area with annual or six-monthly testing, and low-risk area with four-yearly testing^164^. However, high-risk regions must be accurately identified, through analyses of reservoir population densities, spatial layers, and networks of movements and contacts between the different epidemiological groups. The disease epidemiology has to be well understood before robust risk maps can be produced^87^.
4. Trophic chain maintenance and predator restoration are increasingly discussed options in more global and ‘One Health/Eco Health’ approaches for the socio-ecosystem management^117^. These strategies could be active at two levels to protect the ecosystem health. First, predators could preferentially kill infected, weakened prey and hence directly contribute to infection control. This hypothesis has been explored theoretically^165^, but the correlation between predation and disease prevalence is not clear in empirical studies^166^. Second, predators could play the role of a natural barrier between species, decreasing interactions between hosts, and hence pathogen transmission. Empirical studies at the community level are still needed to address these hypotheses. Wolf restoration is advocated in North America to limit brucellosis transmission between bison and elk^167^. Wolf predation may also suppress CWD emergence or limit its prevalence in deer population.^168^. In African ecosystems, lions could limit interactions between cattle and buffalos at the edge of national parks, and thereby hinder FMD transmission^169^. Similarly, restoration of species community for a potential dilution effect is increasingly debated among scientists and stakeholders. Indeed, wildlife species extinction can remove dead-end individuals (i.e., individuals that cannot transmit the pathogen), thus potentially reducing its onward transmission^170^. In parallel, the erosion of livestock genetic diversity resulting from selection to maximize production potentially creates favorable conditions for pathogen adaptations and transmission. The acute threat is that large numbers of animals, potentially a large proportion of a given breed, could die because of a disease or the culling program, thus threatening the livelihood, food security, and rural development of many countries^171^. Diversity helps to make livestock production systems more resilient to shocks.

## Fieldwork constraints and limits

### Field feasibility

Culling a sufficient proportion of individuals in the population is a key determinant of the culling strategy success. However, wildlife culling is often technically difficult (i.e., field access, animal behavior). For instance, the Alpine ibex *(Capra ibex)* culling in the massif du Bargy in France involved carcass removal for incineration by helicopter, in order to avoid environmental and scavenger contamination^89^. Continuous culling is particularly challenging logistically, but also due to the animal adaptation to the culling technique. This can compromise the culling objectives identified through modeling. Culling work can be hampered also by the field teams’ exhaustion, which sometimes results in a progressive decrease in the capture rate^77^. One study estimated that the culling efficiency rate within the Randomized Badger Culling Trial in the UK (see Box 2**)** ranged between 32% and 77%^74^, whereas some dynamic transmission models indicated that 80–100% of animals should be removed^87,90^. Consequently, some researchers concluded that if it is not possible to guarantee a sufficiently high removal of individuals, a non-culling strategy may be more effective in terms of prevalence reduction and less costly than a culling strategy with limited culling efficiency^46^. However, ecological expertise can increase the likelihood of catching animals. This was the case for badgers when researchers found that the weather (rain and temperature) influenced the trapping success^91^.

### Surveillance, diagnosis, and rapid detection

One major limitation of culling, especially in a test-and-cull strategy (see Box 1 and Fig. 1**)**, is the capacity to accurately detect the pathogen or the disease in the targeted population or in individuals. Technical capacities are often suboptimal and diagnostic tests are rarely available for accurate pathogen detection in wildlife, especially for emergent pathogens. CWD control in deer and elk populations in America is a good illustration of the difficulties associated with the diagnosis of infectious diseases in wildlife^92^ (see Supplementary Information—case studies). The appearance of clinical signs in infected animals might take more than two years. Moreover, the available diagnostic tests for urine, saliva or feces are not optimal, with diagnostic sensitivities of 39%, 78%, and 53% respectively, and specificity close to 100% for the three sample types^93,94^. Therefore, an infected individual can remain in the population for a long time without being detected and removed. No prevalence decrease has been observed after 3 years of intensive to intermediate culling in north-western Colorado (USA) between 2001 and 2005^64,92,95^. However, the development of diagnostic tools for the identification of infected animals has been steadily progressing as well as sampling techniques, with a shift from post-mortem to ante-mortem approaches targeting peripheral tissues and bodily fluids^93^.

In many cases, diagnostic tests are based on antibody detection, for example for brucellosis, a chronic disease in the Alpine ibex *(C. ibex)*, and therefore will miss some current active infections. It was estimated that only 51% of all seropositive Alpine ibex *(C. ibex)* individuals excrete the bacteria^96^. The development of rapid tests might lead to animal euthanasia directly in the field after their capture, or after recapture if laboratory analyses are required^97^. However, an undesirable consequence of the use of antibody tests is the removal of individuals that were infected but have recovered and are now immune. Slaughtering seropositive animals may reduce the herd immunity by removing naturally immunized individuals from the population^55^. Moreover, a striking example of the potential consequences of poor test specificity is that 54% of bison individuals culled to control brucellosis in Yellowstone showed no post-mortem sign of brucellosis^55^. Culling many uninfected individuals could jeopardize the persistence of an already small and endangered population (see Supplementary Information—case studies). Therefore, for successful disease control, a test-and-cull approach could be adopted only when good diagnostic tests become available for that pathogen and its infectious period is precisely characterized.

## Predicting the culling response

### Territoriality, behavior modifications, social structure perturbations, and emigration

In natural systems, the social and physiological plasticity of animal populations targeted by a culling campaign can give them a remarkable capacity to adapt and recover after culling^98^. Culling effects go beyond the simple reduction of the population numbers. Animal territoriality is often affected by culling, with crucial consequences on the epidemiology of the targeted pathogen, as recently modeled by Prentice et al.^75^. The behavioral response of badgers to culling in the UK (see Box 2) included increased dispersal within the culled areas. This resulted in an increased overlap of badger social group territories and was associated with increased *M. bovis* prevalence among badgers inside targeted and neighboring social groups^99,100^. Carter et al. showed that the territoriality of badger populations could be perturbed for up to 8 years after the culling action^74^. The same phenomenon of home range perturbation probably occurred in Alpine ibex (*C. ibex*) populations after culling in response to brucellosis re-emergence in adjacent livestock and humans^96^. Therefore, culling modifies the social organization of animal groups, but can also alter the sex ratio, age, and dominance structures and stimulates the survivors’ dispersion in new areas^101^ (Fig. 1c). Moreover, culling can increase stress, leading to immune system depression and higher disease expression^102^.

### Compensatory reproduction, migration, and community species adaptation

In most mammals, population dynamics are driven by density-dependent fecundity and mortality. Because of resource limitations, the demographic parameters related to their reproduction, survival and dispersal vary in function of their own density and also of other species within their ecological community. This results in a regulatory relationship between population growth rate and density in which populations grow when density is below the ecosystem carrying capacity, and decline in the opposite situation (Fig. 1d)^103^. Thus, culling can be partially or fully compensated through these mechanisms, and this could in turn lead to an increase in pathogen transmission^98^. In temperate areas, reproduction seasonality must be taken into account to determine the optimal culling timing. Culling just after the birth pulse and not shortly before the breeding season might limit reproductive compensation phenomena that could increase the number of susceptible individuals and the transmission risks^61^. Culling can also promote immigration into an area with decreased local density and increased resource availability (Fig. 1d)^74,104^. For example, a mass immigration of mice originating from several kilometers away was observed after the culling of deer mice to control Sin Nombre Virus. The entire culled population was replaced within 2 weeks^105^.

## Frameworks for decision-making

**Fig. 2:**
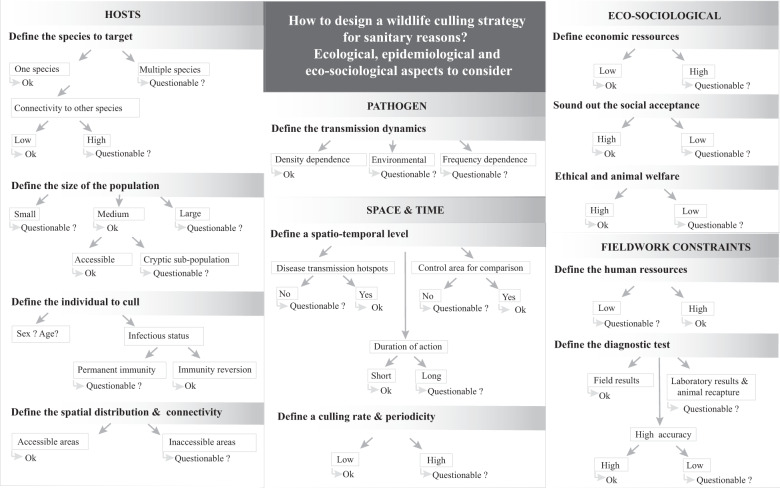
How to design a wildlife culling approach for sanitary reasons: ecological, epidemiological, and eco-sociological aspects. The figure summarizes the road map to follow by taking into account the host, pathogen, space and time, eco-sociological aspects, and fieldwork constraints when a wildlife culling strategy is considered with the aim of controlling an infectious disease. The ‘OK’ tag indicates conditions that when fullfilled, are likely to increase the success of a culling strategy according to the examples found in the literature. When these conditions are not fulfilled (‘Questionable?’), the success of a culling strategy is less likely and good metrics to detect any unexpected result should be put in place.

Before exploring the possible approaches for complementing or replacing culling as a disease control strategy, we will summarize seventeen elements that might increase the likelihood of successful disease control through culling (see Fig. 2). The first six key elements for the efficient implementation of host culling to eradicate a disease were identified by Myers twenty years ago^106^: (1) having the resources to conduct of the whole project; (2) having access to all the necessary areas (public or private); (3) the target species has to be sensitive to the action; (4) post-culling immigration has to be limited; (5) the pathogen has to be detectable at low prevalence; and (6) ecosystem management may be required after the potential eradication of a ‘keystone’ target species. We propose additional elements that should be considered when designing a culling strategy: (7) the target species has to be the only, or at least the primary component of the wildlife chain of the pathogen transmission/reservoir; (8) the target number of individuals to be culled has to be achievable; (9) the target species has to be easy to catch and to cull; (10) infectious individuals should be preferentially removed and immune ones should ideally be left in the system; (11) pathogen transmission should be mainly density-dependent; (12) the areas selected for culling should be areas with the highest disease risk or highly connected to other sensitive areas; (13) control areas without culling need to be considered to evaluate culling effectiveness; (14) the planned culling duration should be reasonable; (15) the culling rate and periodicity have to be achievable; (16) the civil society should understand and approve the action; and (17) enough human resources must be provided in order to avoid field team exhaustion. However, due to the limited success of the culling strategies implemented to date in different species and different ecosystems (Table 1), other policy options should be considered before culling. These options are summarized and compared with culling in Box 4.

## Conclusion and recommendations

Effective culling strategies for wildlife pathogens require a number of conditions that are challenging to meet when all constraints are considered (see Fig. 2). Importantly, culling can lead to counterintuitive and detrimental outcomes, for instance higher disease incidence in some areas^36,37^. Therefore, a decreased disease risk is far from being a guaranteed result with culling.

Ecological, sociological, and epidemiological contexts have to be fully considered to inform the choice, design, and the final decision on such a strategy. Before choosing culling, it is particularly important to consider all involved host populations and to evaluate their contribution to pathogen maintenance and transmission^29,41^. This review demonstrates that culling wildlife reservoir populations might ultimately exacerbate the spread of a given disease, depending on the situation^54,72,73,81,107^. Such negative effects are difficult to anticipate due to the complexity of the relationships between host density, host contact rates, and disease incidence. In addition, wildlife populations are often inaccessible, which makes their demographic and epidemiological characterization difficult. Before wildlife culling is undertaken, pathogen transmission pattern, host contact pattern, regulatory processes, seasonality, spatial structure, and environmental sources of infection should be precisely understood^46,80^. Today, scientists call for the creation of evidence-based syntheses for policy makers that are inclusive, rigorous, transparent, and accessible: “Rapid synthesis can respond more tactically to emergencies or, more commonly, to the day-to-day business of government. It can involve rapid evidence assessments, which are more targeted than a systematic review, with more-restricted search terms, evidence-gap maps and semi-structured interviews - techniques which ensure that more voices and views are considered and weighed, and which go beyond what a scientist would typically consider a ‘review’”^108^.

**Fig. 3:**
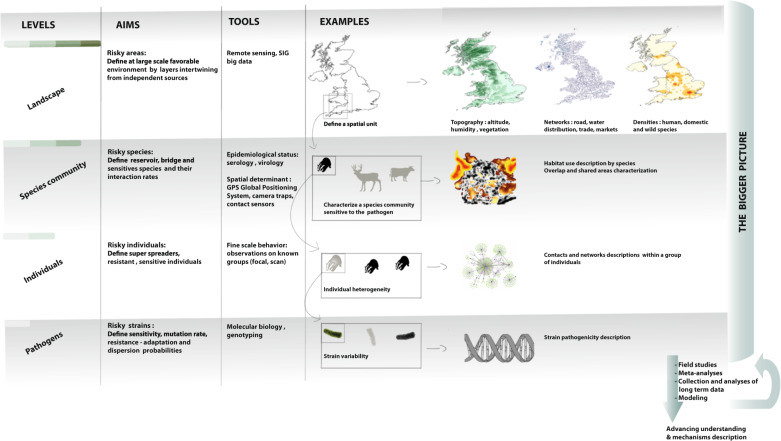
Toolbox for disease management in wildlife population(s): ecosystem levels relevant to disease control. Understanding, controling and maintaining surveillance systems on infectious diseases in wildlife requires that research activities from different disciplines should be set up simultaneously to study pathogens, indidividuals, species communities and landscapes. The global aim is to get a bigger picture of the socio-ecosystem dynamics with frequent back and forth from field activities, bibliography syntheses and modelling outputs.

Management strategies should be multifaceted and adaptive through space and time^109^. The design of an efficient and acceptable culling strategy would require a systems-based approach built on the mechanistic understanding of the system key components and their interactions, giving careful consideration particularly to the range of species involved. Surveying a wide network of transmission hubs will facilitate the identification of major and minor host species, and will give a greater understanding of the transmission risks (Fig. 3). Non-invasive methods for pathogen detection in wildlife are needed. The recent development of techniques for specimen collection and pathogen detection from the environment^110^, oral fluids (i.e., saliva^111^), feces^112^, and blood from blood-sucking flies^113^ constitutes an important scientific breakthrough. It has the potential to open the black box of wildlife infectious disease dynamics. These new approaches could dramatically decrease the costs related to wildlife fieldwork and long-term longitudinal surveys of populations from different species and/or in risky areas. They could also allow increasing the spatial range of epidemiological surveys, and thus the likelihood of catching pathogens for genetic analyses and for understanding the evolution of pathogen transmission among hosts^88^.

When efficient (inducing long-term immunity) and easily administrable vaccines are available, vaccination may be a more appropriate option, although the cost will be an important factor to consider. Immunization results in the reduction in the frequency of susceptible individuals in the population, whatever the pathogen transmission mode. However, tools for the differential diagnosis between vaccine- and infection-induced antibodies are needed to monitor the natural infection dynamics in the population.

Finally, wildlife culling should only be considered as an option for disease control by reducing the pathogen’s ecological niche^114^, in combination with the complementary and alternative approaches detailed in Box 4^115^.

The study of ecosystems is likely to provide insights into the consequences of culling and other disease control policies on host behavior and up to landscape-level patterns of transmission risk. It should stimulate the cooperation of all actors (policy makers, researchers, public and veterinary health managers, and ecosystem and biodiversity managers), and lead to proactive, rather than reactive approaches to disease control^87^ (Fig. 3). Particularly, risk maps that integrate multiple layers^116^ could be produced for surveillance planning and effort monitoring. When pathogen dynamics are poorly understood, adaptive management approaches should be adopted^64^, in which continuous feedback between modeling and empirical studies, including epidemiological and demographic analyses, allows progressive improvements in the estimation of key parameters, scientific hypothesis testing, and diagnosis.

To conclude, the control of infectious diseases in wildlife is a complex subject for which no magic bullet exists. However, it is crucial to highlight the importance of maintaining long-term data collection and surveillance systems with the objective of monitoring population dynamics, detecting rapidly emergent events, and avoiding endemicity. It is also important to maintain the biodiversity and specific richness of the wild and domestic compartments, two key drivers of resilient socio-ecosystems^117^.

## Supplementary information

Supplementary Information
