# Supplementary material for: A systemic approach to assess the potential and risks of wildlife culling for infectious disease control
Source: Commun Biol. 2020 Jul 7;3:353. doi: 10.1038/s42003-020-1032-z (PMC7340795; doi:10.1038/s42003-020-1032-z)
Supplement: Supplementary file 1 — Supplementary Information [file 42003_2020_1032_MOESM1_ESM.pdf]

Supplementary Information for 'Potential and practice of wildlife culling for disease control': Article selection for the review and descriptive statistics

Two rounds of article selections were done for gathering the best information on the research question.

1/Article and data collection

The articles selected for the review were identified using the Web of Knowledge database. The search was restricted to articles and reviews in English, classified in the "infectious diseases" subject area. All entries that were also classified in the "plant science" subject area were excluded. A number of keywords were used to identify reports of anthropogenic changes in mortality or population density of wild animals for health and non-health related reasons: "pest control", "pest management", "rodent control" and specific methods ("cull", "harvest", "hunt", "fishing", "game cropping", "insecticide", "rodenticide"). These keywords were used in conjunction with a filter for population reduction or control. Additional searches (within the classifications specified above) were also performed to identify articles that included the phrase "population perturbation" or that used "unexpected" in the title. An initial review of the obtained titles and abstracts allowed excluding studies not related to infection in wild animals or in artificial settings. This process was complemented with suggestions for specific infections or case studies from expert informants and from textbooks on the management of wildlife diseases. Furthermore, specific efforts were made to identify case studies across a wide range of wildlife (terrestrial, aquatic and airborne).

2/ Database development

After the first round of article selection, 475 abstracts from 1935 to 2011, were read and different parameters were recorded in order to achieve a large overview of the topic without any a priori focus: "Pathogen /Parasite Disease"; "Association to humans/ livestock disease"; "Intervention on target species"; "Intervention on a secondary target species"; "Interference "; "Location"; "Date"; "Target species Disease Outcome"; "Method of culling"; "Duration"; "Range/Scale"; "Other activity in addition to culling methods"; "Known density effects"; "Disease dynamic"; "Type of Transmission

route”; “ Result in controlled population”; “Result in other wildlife species”; “Result in humans/ livestock”; “Evidence available”; “Limitations /caveats”; “Explanations”.

The distribution of abstracts recorded was as follows: 332 abstracts from journals, 40 from book chapters, 82 from books, and 21 from reports. A database of 217 items was built from this first round of literature review, and the structure of the article was drafted. After the first review draft was written, regular literature updates were carried out in the Web of Knowledge database with the following key words: ‘infectious diseases’ AND ‘culling’ AND ‘wild’ until January 2019.

The final database included 466 articles from 1992 to 2018. The selection criteria for articles based on their abstracts were: exclusion of theoretical papers and of articles on insect control; and inclusion of articles mainly on mammals, of articles that prioritize empirical data and experiments, of articles in which culling is a central topic and not just mentioned in Introduction or Discussion.

3/ Descriptive analyses

The descriptive analyses of the collected literature data showed that research concerning the understanding and the control of wildlife diseases has substantially increased in the 2000s (Fig. 1), mainly in the veterinarian sector (supplementary table 1). By selecting journals that were mentioned at least 5 times in our database, 43% of the selected articles were published in veterinary journals, 32% in ecological or wildlife journals, 14% in epidemiological journals, 9% in generalist journals, and 3% in agricultural journals.

Furthermore, some diseases or animal species were more studied. Counting the occurrence of different words in the title of the selected articles showed that “badgers” was mentioned 91 times in our database, “bovine tuberculosis” 51 times, “brucellosis” 17 times, “bison” 20 times, “conservation” 9 times, “evolution”/“evolutionary” 6 times, and “ecology” also 6 time.

Ecology and evolution were not frequently mentioned in our database, suggesting that the pathogen adaptation to new environments or hosts after a perturbation, such as culling, is understudied. This scientific gap emphasizes the importance of building bridges between disciplines, as proposed by the “one health/eco health” framework, given the complexity of the subject.

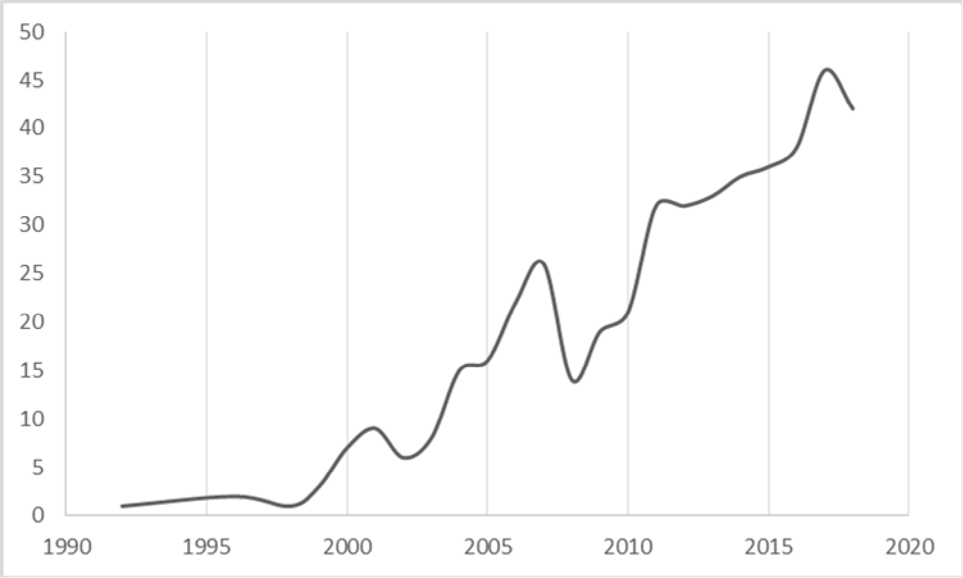

Supplementary figure 1: Number of articles referring to infectious diseases, culling and wildlife in the Web of Knowledge database from 1992 to 2018.

Names of journals recorded in the database from 1992-2018	Number of articles
VETERINARY RECORD	55
JOURNAL OF WILDLIFE DISEASES	18
PREVENTIVE VETERINARY MEDICINE	15
JOURNAL OF APPLIED ECOLOGY	13
PLOS ONE	12
JOURNAL OF WILDLIFE MANAGEMENT	11
TRANSBOUNDARY AND EMERGING DISEASES	11
VETERINARY MICROBIOLOGY	10
JOURNAL OF ANIMAL ECOLOGY	9
EUROPEAN JOURNAL OF WILDLIFE RESEARCH	8
JOURNAL OF AGRICULTURAL & ENVIRONMENTAL ETHICS	8
PROCEEDINGS OF THE ROYAL SOCIETY B-BIOLOGICAL SCIENCES	8
VETERINARY PARASITOLOGY	8
PARASITOLOGY	6
REVUE SCIENTIFIQUE ET TECHNIQUE-OFFICE INTERNATIONAL DES EPIZOOTIES	6
VETERINARY JOURNAL	6
AVIAN DISEASES	5
ECOLOGICAL APPLICATIONS	5
MAMMAL REVIEW	5
VACCINE	5
WILDLIFE RESEARCH	5
ZOONOSES AND PUBLIC HEALTH	5

Supplementary table 1: Number of articles recorded in our database according to the title of the journal from 1992 to 2018. Veterinarian journals are highlighted in orange, ecological journals in green, generalist journals in blue, epidemiological journals in yellow, and agricultural journal in grey.

Supplementary Information: Case studies

1/ Case study of the eradication efforts of endemic pathogens at the wild/domestic interface: veterinary, public health, and conservation issues.

Brucellosis (Brucella abortus): cattle, bison (Bison bison) and elk (Cervus elaphus) - United States of America

Brucellosis is caused by *Brucella abortus* that was introduced in the United States of America (USA) via European livestock. *B. abortus* was first detected in bison in Yellowstone National Park in 1917 [1] and in elk in 1930. Human infection (painful, debilitating, and chronic) is usually due to the consumption of unpasteurized dairy products and/or the handling of infected material (e.g. aborted fetuses). Brucellosis eradication campaigns, initiated in 1934 through cattle vaccination and the slaughter of entire cattle herds where positive animals were detected considerably reduced the outbreak frequency in the USA.

Brucella bacteria are well adapted to ungulates and in these hosts, they cause slight morbidity or mortality (e.g. abortion of fetuses) [2]. In wildlife and cattle, uninfected animals contract the disease primarily by licking birth exudates. Grazing on contaminated forage is a less likely intraspecies transmission route. The density of infected animals and contact rates between infectious and susceptible individuals influence the transmission rates [3]. Elk and bison are currently assumed to be the primary wildlife reservoirs of brucellosis in the Yellowstone ecosystem. Free-ranging elk have been implicated as the source of multiple brucellosis transmission events to cattle, due to the lack of contact between bison and infected cattle herds. However, despite the lack of evidence on the role of bison in brucellosis transmission to cattle, a quarter of the Yellowstone bison population was culled in 2007 with the objective of eradicating brucellosis in wildlife. This expensive test-and-cull operation failed to limit the spread of brucellosis in the Yellowstone area for several reasons [4]. First, *B. abortus* can survive in grazing land for 5 years, providing an important route of transmission through the environment, independently of host densities. Second, elk (in which prevalence varies between 1 and 37% depending on the area) are rarely shot outside the Yellowstone National Park. As a

consequence elk maintain the transmission chain between wildlife reservoir and livestock populations [4]. Finally, as *B. abortus* could persist in small host populations (e.g. less than 200 individuals) [5], disease eradication through culling would require the removal of a considerable fraction of the reservoir host population. This might drive the wildlife reservoir population host to the brink of extinction and generate conservation concerns. Transmission models showed that vaccination of 40-50% of the bison population would have a significant effect. However, this objective is difficult to achieve with intradermic vaccines [4]. A recent genomic study revealed that elk are the source of livestock infections and that control measures in bison are unlikely to affect the dynamics of unrelated strains circulating in nearby elk populations [6].

2/ Case studies on the eradication of emergent pathogens in wildlife, a conservation issue:

- ***Canine distemper virus in the Serengeti ecosystem***

The canine distemper virus killed one third of the Serengeti lion population (*Pathera leo*) in 1994 [7]. Domestic dogs *(Canis lupus)* were initially identified as the virus reservoir species due to genetic similarity between virus strains identified in lions and dogs. Additional studies highlighted the importance of other wild carnivores, such as jackals (*Canis spp.*) and hyenas (*Crocuta crocuta),* in the transmission dynamics and interaction network [8]. Years later, a study described a ‘meta-reservoir’ that encompassed multiple connected carnivore populations (domestic and wild), thus posing significant challenges for control [9, 10]. Furthermore, a retrospective study of seven epidemics from 1987-2007 showed that the excess mortality in lions also implicated co-infection by pathogens transmitted by vectors (*babesia* bacteria transmitted by ticks). Following extreme dryness and heavy rains, the tick density increased dramatically as well as the tick infestation rate in buffalos, the main preys of lions [11]. This example illustrates the challenges in controlling pathogens that can persist in multiple host species and interact with other pathogens in the context of environment perturbations, such as climate change.

- ***Chronic Wasting Disease: mule deer (Odocoileus hemionus), white-tailed deer (Odocoileus virginianus), elk (wapiti, Cervus canadensis), and moose (Alces alces) - North America***

Chronic wasting disease (CWD) is an emerging neurodegenerative prion disease that affects the North American mule deer, white-tailed deer, elk (wapiti,), and moose. Other prion diseases that affect a large range of hosts are known, including Bovine Spongiform Encephalopathy (BSE) in cattle, scrapie in sheep and goats, and Creutzfeldt-Jakob Disease (CJD) in humans [12]. CWD origin is unknown. It was detected for the first time in Colorado (USA) in 1967, and continues to emerge and spread in cervid populations throughout the USA and Canada. It is still confined to North America [13]. In March 2020, CWD in free-ranging deer, elk and/or moose was reported in at least 24 states in continental USA, and in two provinces in Canada. CWD has been detected also in reindeer and moose in Norway, Finland and Sweden and a small number of imported cases have been reported in South Korea. The disease was also found in farmed deer and elk [14]. The prevalence in mule deer populations reached almost 50% in endemic areas of Wyoming, and 15% in Colorado. It ranges between 0 and 5% in Wisconsin and other areas.

CWD directly threatens wildlife populations and also affects hunting, tourism, agricultural industries and potentially human health, even if its long-term effects remain unclear. Horizontal transmission through contact with urine, feces, saliva and blood of infectious individuals has been described. In addition, the environment could be a natural reservoir [15], thus hampering attempts to eradicate the disease or control its spread.

Non-selective culling to reduce the overall deer populations and selective culling of infected individuals to decrease their frequency have been used to control CWD in free-ranging populations [16]. However, selective culling effectiveness is limited because it can take more than 2 years for an infected animal to show clinical signs [17]. After 3 years of intensive culling and after 6–7 years of low to intermediate culling of mule deer in Colorado [18], CWD prevalence was not reduced [19]. This could be explained by the fact that CWD has been long established in the population and by the pathogen persistence in the environment. Indeed, prions could persist in pastures containing infected carcasses for at least 2 years [15, 20]. However, culling may be effective in areas where the disease

has just emerged, as observed in the New York and Minnesota regions [13]. Nevertheless, modeling indicates that the time needed to reach eradication with intensive culling ranges from several years to centuries [21]. Despite relatively low epidemic growth rates, the basic reproduction number (R_0) might be much higher than expected under the direct-transmission paradigm because the infectious period can vastly exceed the host life span. Therefore, culling becomes increasingly less effective as CWD epidemic progresses [15].

Bibliography

1. Cheville, N.F., D.R. McCullough, and L.R. Paulson, *Brucellosis in the Greater Yellowstone Area.* Brucellosis in the Greater Yellowstone Area., 1998(Journal Article): p. xvii + 186 pp.
2. OIE, *Brucellosis (Brucella abortus, B. Melitensis and B. Suis) (Infection with B. abortus, B. Melitensis and B. Suis).OIE Terrestrial Manual 2016*. OIE Terrestrial Manual 2016. Vol. OIE Terrestrial Manual 2016. 2016.
3. Proffitt, K.M., P.J. White, and R.A. Garrott, *Spatio-temporal overlap between Yellowstone bison and elk – implications of wolf restoration and other factors for brucellosis transmission risk.* Journal of Applied Ecology, 2010. **47**: p. 281–289.
4. Bienen, L. and G. Tabor, *Applying an ecosystem approach to brucellosis control: can an old conflict between wildlife and agriculture be successfully managed?* Frontiers in Ecology and the Environment, 2006. **4**(6): p. 319-327.
5. Dobson, A. and M. Meagher, *The population dynamics of brucellosis in the Yellowstone National Park.* Ecology, 1996. **77**(4): p. 1026-1036.
6. Kamath, P.L., et al., *Genomics reveals historic and contemporary transmission dynamics of a bacterial disease among wildlife and livestock.* Nature Communications, 2016. **7**: p. 11448.
7. Roelke-Parker, M.E., et al., *A canine distemper virus epidemic in Serengeti lions (Panthera leo).* Nature, 1996. **379**(6564): p. 441-5.
8. Craft, M.E., et al., *Distinguishing epidemic waves from disease spillover in a wildlife population.* Proceedings of the Royal Society B: Biological Sciences, 2009. **276**(1663): p. 1777-1785.
9. Prager, K.C., et al., *Rabies Virus and Canine Distemper Virus in Wild and Domestic Carnivores in Northern Kenya: Are Domestic Dogs the Reservoir?* Ecohealth, 2012. **9**(4): p. 483-498.
10. Viana, M., et al., *Dynamics of a morbillivirus at the domestic-wildlife interface: Canine distemper virus in domestic dogs and lions.* Proc Natl Acad Sci U S A, 2015. **112**(5): p. 1464-9.

11. Munson, L., et al., *Climate Extremes Promote Fatal Co-Infections during Canine Distemper Epidemics in African Lions.* PLOS ONE, 2008. **3**(6): p. e2545.

12. Gross, J.E. and M.W. Miller, *Chronic wasting disease in mule deer: disease dynamics and control.* Journal of Wildlife Management, 2001. **65**(2): p. 205-215.

13. Saunders, S., S. Bartelt-Hunt, and J. Bartz, *Occurrence, Transmission, and Zoonotic Potential of Chronic Wasting Disease.* Emerging Infectious Diseases, 2012. **18**(3): p. 369-376.

14. CDC. *Chronic Wasting Disease (CWD) Occurrence.* https://www.cdc.gov/prions/cwd/occurrence.html. 2017.

15. Almberg, E.S., et al., *Modeling Routes of Chronic Wasting Disease Transmission: Environmental Prion Persistence Promotes Deer Population Decline and Extinction.* PLOS ONE, 2011. **6 (5)**(5): p. e19896.

16. Williams, E.S. and M.W. Miller, *Chronic wasting disease in deer and elk in North America.* Revue Scientifique Et Technique De L Office International Des Epizooties, 2002. **21**(2): p. 305-316.

17. Bunk, S., *Chronic wasting disease - Prion disease in the wild.* Plos Biology, 2004. **2**(4): p. 427-430.

18. Miller, M.W. and M.M. Conner, *Epidemiology of chronic wasting disease in free-ranging mule deer: Spatial, temporal, and demographic influences on observed prevalence patterns.* Journal of wildlife diseases, 2005. **41**(2): p. 275-290.

19. Conner, M.M., et al., *A meta-BACI approach for evaluating management intervention on chronic wasting disease in mule deer.* Ecological Applications, 2007. **17**(1): p. 140-153.

20. Miller, M.W., et al., *Environmental Sources of Prion Transmission in Mule Deer.* Emerging Infectious Diseases, 2004. **10**(6): p. 1003-1006.

21. Wasserberg, G., et al., *Host culling as an adaptive management tool for chronic wasting disease in white-tailed deer: a modelling study.* Journal of Applied Ecology, 2009. **46**(2): p. 457-466.

203
